# Supplementary material for: Identification of Potent Zika Virus NS5 RNA-Dependent RNA Polymerase Inhibitors Combining Virtual Screening and Biological Assays
Source: Int J Mol Sci. 2023 Jan 18;24(3):1900. doi: 10.3390/ijms24031900 (PMC9915956; doi:10.3390/ijms24031900)
Supplement: Supplementary file 1 [file ijms-24-01900-s001.zip › ijms-2143487-supplementary.pdf]

Supplementary material

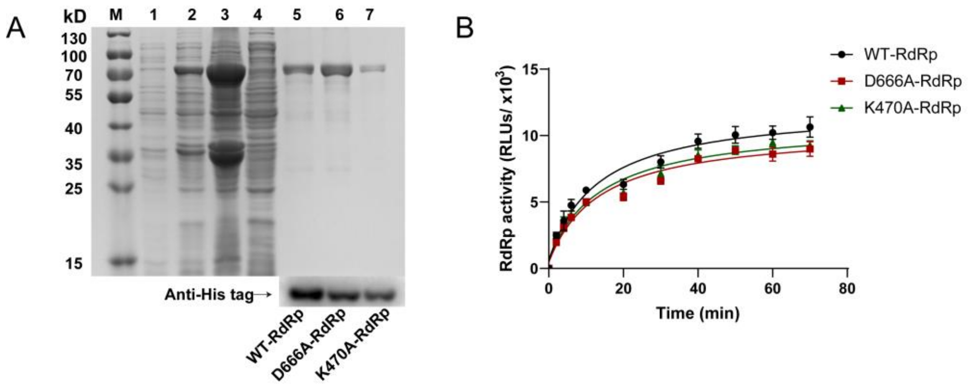

Figure S1. Purification and characterization of RdRp. (A) SDS-PAGE (top) and Western blot (bottom) analysis of RdRp. Lane M, protein marker. Lane 1, total cellular proteins in pET30a (Transetta). Lane 2, total cellular proteins in pET30a-RdRp (Transetta); Lane 3, sediment of cellular lysate in pET30a-RdRp (Transetta). Lane 4, supernatant of cellular lysate in pET30a-RdRp (Transetta). Lane 5-7, purified proteins of RdRp, D666A-RdRp and K470A-RdRp. Western blot results of them are on the bottom. (B) The catalytic activity of RdRp, D666A-RdRp and K470A-RdRp.
